# Genetic variation of maturity groups and four *E* genes in the Chinese soybean mini core collection

**DOI:** 10.1371/journal.pone.0172106

**Published:** 2017-02-16

**Authors:** Jicun Li, Xiaobo Wang, Wenwen Song, Xinyang Huang, Jing Zhou, Haiyan Zeng, Shi Sun, Hongchang Jia, Wenbin Li, Xinan Zhou, Suzhen Li, Pengyin Chen, Cunxiang Wu, Yong Guo, Tianfu Han, Lijuan Qiu

**Affiliations:** 1 MOA Key Laboratory of Soybean Biology (Beijing), Institute of Crop Science, Chinese Academy of Agricultural Sciences, Beijing, China; 2 Jining Academy of Agricultural Sciences, Jining, Shandong, China; 3 School of Agronomy, Anhui Agricultural University, Hefei, China; 4 Heihe Branch of Heilongjiang Academy of Agricultural Sciences, Heihe, China; 5 College of Agronomy, Northeast Agricultural University, Harbin, China; 6 Oil Crops Research Institute, Chinese Academy of Agricultural Science, Wuhan, China; 7 Department of Crop, Soil and Environmental Sciences, University of Arkansas, Fayetteville, Arkansas, United States of America; National Institute for Plant Genome Research, INDIA

## Abstract

The mini core collection (MCC) has been established by streamlining core collection (CC) chosen from China National Genebank including 23,587 soybean (*Glycine max*) accessions by morphological traits and simple sequence repeat (SSR) markers. Few studies have been focused on the maturity that has been considered as one of the most critical traits for the determination of the adaptation-growing region of the soybean. In the current study, two hundred and ninty-nine accessions of MCC planted for two years at four locations namely in Heihe, Harbin, Jining and Wuhan cities in China were used to assess the variation of maturity in MCC and identify the integrated effect of 4 *E* loci on flowering and maturity time in soybean. Forty-two North American varieties served as references of maturity groups (MG). Each accession in MCC was classified by comparing with the MG references in the days from VE (emergence) and physiological maturity (R7). The results showed that MCC covered a large range of MGs from MG000 to MGIX/X. Original locations and sowing types were revealed as the major affecting factors for maturity groups of the MCC accessions. The ratio of the reproductive period to the vegetative period (R/V) varied among MCC accessions. Genotyping of 4 maturity genes (i.e. *E1*, *E2*, *E3* and *E4*) in 228 accessions indicated that recessive alleles *e1*, *e2*, *e3* and *e4* promoted earlier flowering and shortened the maturity time with different effects, while the dominate alleles were always detected in accessions with longer maturity. The allelic combinations determined the diversification of soybean maturity groups and adaptation to different regions. Our results indicated that the maturity of Chinese soybean MCC showed genetic diversities in phenotype and genotype, which provided information for further MG classification, geographic adaptation analysis of Chinese soybean cultivars, as well as developing new soybean varieties with adaptation to specific regions.

## Introduction

China has the richest germplasm resources of soybean in the world[1]. More than 23,587 soybean accessions had been collected from 29 provinces of China until 2007[2]. Evaluation of soybean germplasm collection is crucial for the selection of elite parents, identification of desirable alleles, as well as breeding of new varieties[3–5]. However, it is still a challenge to evaluate the genetic characterization of the accessions in the Genebank due to abundant resources of the germplasm. Frankel and Brown (1984) firstly proposed the concept of “core collection” (CC), defined as a sub-set of accessions (about 10% of the original size) selected by an optimal sampling method, to represent the maximum genetic diversity of the whole collection[6]. Afterwards, the development of CC has proven to be a reasonable approach to explore the variations from genetic resources[7–9]. Previously, a soybean CC with a rational size containing 472 accessions has been built based on the simple sequence repeat (SSR) marker data and agronomic traits[10]. As the accessions number of the CC is too large for the replicated evaluations at different locations, more manageable mini core collections (MCCs) of soybean have been developed based on further streamlining of the CC scale (10% of the CC), which represented 94.5% of the phenotypic diversity and 63.5% of the genetic diversity of the whole collection, respectively[11–12]

Our previous data showed the soybean accessions in MCC could be used for basic studies including gene discovery, allele mining, marker-trait associated analysis, and gene functional analysis[13]. For example, 70 SSR markers were used to evaluate a set of 96 wild soybean accessions in MCC, which indicated that a total of 1,278 alleles were identified with an average of 18.2 alleles per locus[14]. In addition, backcross introgression lines developed from soybean cultivars in the MCC were used to identify QTLs related to cold and drought stress[15–16]. Moreover, Guo and Qiu (2013) developed the allele-specific marker for the selection of allelic distributions of flavonoid 3′-hydroxylase (*GmF3'H*) and flavonoid 3′, 5′-hydroxylasegenes (*GmF3'5'H*) genes in 170 soybean accessions in MCC[17]. Furthermore, MCC also provided trait-specific resources for soybean improvement programs. For instance, resistance to soybean mosaic virus (SMV) was evalauted using inoculation of four Chinese SMV strains in soybean CC from Southern China [18]. Also, MCC contained a wider range of protein subunits of 11S and 7S than common cultivars, which were the major components of seed storage protein in soybean[19]. Moreover, in a study using the landraces of MCC for the identification of allele variations of soybean stem growth habit gene *GmTFL1*, the genetic diversity and geographic distribution of the four *Gmtfl1* alleles revealed that artificial selection for soybean determinacy occurred at early stages of landrace dissemination. Whereas, only one *GmTfl1* allele was screened in the wild soybeans, indicating the effects of genetic bottlenecks created by germplasm introduction and modern breeding[20]. Therefore, such collection has been well acknowledged as an ideal candidate for the identification of trait-specific accessions, gene discovery and molecular breeding in soybean.

Length of growing period or maturity is an important trait of crops as it determines the geographical adaptation of a variety[21–24]. In North America, soybean had been classified into 13 MGs, which were designated by Roman numerals, starting with “000” MG adapted to long days in Canada, and ending with “X” MG adapted to short days in Southernmost areas of the US[25]. Zhang et al. (2007) examined the adaptation area of different MG soybean varieties in the US, which showed that soybeans of MG0 to MGVI were mainly grown in the major producing areas of the US, and MGVII and MGVIII were currently cultivated in a limited region in the southern states[26]. In Argentina, soybeans of MGII to MGIX could be grown under suitable environments from September to February each year[27]. Alliprandini et al. (2009) developed an efficient method for assigning relative MGs to the commercial cultivars based on the evaluation of the maturity stability of 48 Mid-Western and 40 southern Brazilian commercial cultivars ranging from MGVI to MGVIII at 15 locations[28]. In China, extensive studies have also carried out to categorize soybean accessions into different MG groups based on environment and planting patterns [29–35]. For example, Hao et al. (2003) identified 12 soybean MGs (C1-C12) by planting 96 soybean varieties at 28 locations, but such classification was not linked with the MG system in North America[33]. Gai et al. (2001) analyzed the maturity time of 264 Chinese soybean landraces under natural and extended day-length conditions in Nanjing city and confirmed the presence of soybeans of MG000 to MGIX in China when comparing with the 48 varieties of 13 MGs from the US[4]. Additionally, the geographic distribution scheme of soybean MGs was proposed in China. The same classification will be beneficial for the comparison and exchange of soybean germplasm in a national and international scale.

Flowering and maturity of soybean are controlled by the major genes, and to date, at least nine maturity loci (*E1*-*E8*, and *J*) have been identified[36–42]. Up to now, extensive studies have been performed on the identification and characterization of *E1-E4* genes at a molecular level[43–46]. The *E1* gene encoded a transcription factor with a putative nuclear localization signal and a B3-related domain[46]. Allelic variations in the *E1* gene included single nucleotide polymorphism (SNP) (*e1-as*) or single base deletion (*e1-fs*) at coding sequence and a null allele (*e1-n1*)[46] For the *E2* gene, an orthologue of *Arabidopsis* flowering gene *GIGANTEA* was found to be linked to the *E2* locus, and a nonsense mutation resulted in premature stop codon was identified in the *e2-ns* allele[45]. The phytochrome genes *GmPhyA3* and *GmPhyA2* were reported to localized in the *E3* and *E4* loci, respectively[43, 44]. Meanwhile, at least six alleles of *E3* were detected using sequencing technique, while at least five alleles were detected for *E4* [47]. Genotyping by sequencing (GBS) approaches were also used to develop tools for breeders to rapidly identify alleles present in their germplasm at the recently cloned maturity loci[48]. Although four known maturity loci (i.e. *E1*-*E4*) contribute to our understanding on the mechanism of flowering and maturity, genotypes at the *E* loci and the relationship with maturity group in Chinese soybean MCC are still not well defined.

In this study, 299 Chinese soybean MCC and 42 MG representative cultivars from the US were tested to classify the maturity groups of MCC in China. In addition, genotyping of four maturity genes was performed in accessions of MCC. These results could enrich the understanding on the variations in MCC and contribute to the prediction of varieties adaptability in suitable geographical regions efficiently.

## Materials and methods

### Plant materials

The Chinese soybean MCC consisted of 299 accessions selected from the whole soybean collection of the National Genebank of China was used in this study[12]. Forty-two varieties of MGs (MG000-MGVIII) used as MG references were introduced from the United States (Table 1). Reference varieties were selected for each MG, including two early and two late accessions from each group except for MG000 with only one early and one late accessions included.

**Table 1:** Traits of the MG reference varieties planted in spring in Heihe, Harbin, Jining and Wuhan in China.

Variety (PI No.)	MG	Days from emergence (Ve) to physiological maturity (R7)			
		Jining	Heihe	Harbin	Wuhan
Maple Presto (PI548594)	000	65.3±0.67	81.97±0.25		
OAC Vision (PI567787)	000	66.5±0.47	87.83±0.18		
Canatto (PI548648)	00	61.3±0.71	92.00±0.41		
Maple Ridge (PI548596)	00	67.4±0.36	96.3±0.34		
Jim (PI602897)	00	78.0±1.86			
Glacier (PI592523)	00	70.1±0.37	95.17±0.15		
MN0201 (PI629004)	0	70.1±0.96	99.40±0.12	96.5±0.17	
Traill (PI596541)	0	74.0±0.92	99.13±0.13	98.7±0.53	
Surge (PI599300)	0	80.4±1.04		105.0±0.33	
MN0901 (PI612764)	0		115.37±0.11	110.0±0.00	
Harlon (PI548571)	I	88.3±3.33			
Haroson (PI548641)	I	78.3±1.03		99.0±0.00	
NE1900 (PI614833)	I	91.8±0.34		111.5±0.50	
Titan (PI608438)	I	86.2±1.15		107.5±0.83	
Holt (PI561858)	II	97.5±0.50			
OAC Talbot (PI567786)	II	88.8±0.91			
Amcor89 (PI546375)	II	100.1±0.50			
Flint (PI595843)	II	102.2±0.59			
Burlison (PI533655)	II	100.8±0.62			
Athow (PI595926)	III	102.0±0.48			
Zane (PI548634)	III	104.7±0.32			
Macon (PI593258)	III	106.4±0.48			
Saline (PI578057)	III	118.6±1.49			
NS93-4118 (PI614155)	IV	110.6±0.67			
Flyer (PI534646)	IV	115.1±0.65			
TN4-94 (PI598222)	IV	124.6±0.47			
Manokin (PI559932)	IV	126.6±0.24			
Nathan (PI564849)	V	120.2±1.29			
Holladay (PI572239)	V	129.9±0.25			
Lonoke (PI633609)	V	134.1±0.35			
Rhodes (PI561400)	V	133.5±0.40			
Desha (PI633610)	VI	141.9±1.23			141.6±0.86
Dillon (PI592756)	VI	135.0±1.22			142.1±0.93
NC-Roy (PI617045)	VI	152.8±0.50			148.0±1.64
Musen (PI599333)	VI	153.1±0.30			156.9±1.44
Stonewall (PI531068)	VII	154.1±0.59*			153.6±0.89
Benning (PI595645)	VII	154.3±0.13*			154.7±0.98
Santee (PI617041)	VII	154.0±0.22*			156.5±0.70
Hagood (PI555453)	VII	163.7±0.13*			161.3±0.95
Motte (PI603953)	VIII	159.0±0.00*			163.1±0.39
Foster (PI548970)	VIII	159.0±0.00*			158.5±1.46
Crockett (PI535807)	VIII	144.6±1.60*			
Dowling (PI548663)	VIII	158.0±0.00*			150.4±0.74

*Data were estimated according to soybean mature degree at harvest.

### Field experiments

Field experiments were conducted at four locations: Heihe (50.15°N) and Harbin (45.68°N) in Heilongjiang Province, Jining (35.46°N) in Shandong Province, and Wuhan (30.63°N) in Hubei Province. All accessions were planted in Jining city during 2010 and 2011 with a planting date of May 4. In order to classify the MG accurately, some cultivars were planted at the locations in 2011, and finally normal maturity was achieved for each cultivar. Twelve early maturing varieties of MCC and the US varieties belonging to. MG000, MG00, and MG0 were planted in Heihe on May 9, 2011. Fifteen relative early-maturing MCC varieties and US varieties of MG0 and MGI were planted in Harbin on May 4, 2011. Fifty-seven late-maturing MCC soybeans and US varieties of MGVI, MGVII, and MGVIII were planted in Wuhan on April 29, 2011. All accessions were bunch-planted in plots of 40 cm in diameter and 50 cm apart. Three replications were designed, and five plants were finally selected from each bunch for further analysis. Seed resource for each variety was the same for all locations, years and replications. Standard local practices for soybean production were used to manage the experimental plots. Data were collected from 15 plants of 3 bunches for each accession in each location. Number of days from the emergence (VE) to the first flowering (R1), from the VE to the physiological maturity (R7) were measured. The maturity was assessed when one pod in the main stem reached its final pod color[49].

### Genotyping the maturity genes

Genomic DNA was extracted from leaf tissues of each accession using a modified Cetyltrimethyl ammonium bromide (CTAB) method[50]. The SNPs between different alleles of *E1*, *E2*, *E3*, and *E4* genes were chosen according to the previous description by Tsubokura et al. (2014)[47]. The genotype of each allele was analyzed using the Sequenom MassARRAY iPLEX platform[51]. The resulting data was analyzed using the MassARRAY Typer 4.0 Analyzer software. The alleles of each maturity gene were identified by the SNPs.

### Data analysis

Average days to maturity of the US reference varieties in each MG were used to determine the range of each group. The median of neighboring MGs was designed as the threshold of the two groups. Relative maturity of Chinese soybean MCC was classified according to the range of each group[34]. The ratio of reproductive (R) growth and the vegetative (V) growth duration time (R/V) was calculated according to the R1 and R7[52].

## Results

### Growth duration ranges of the maturity group reference varieties from the US

The days to maturity of soybean MG reference cultivars planted at Jining, Heihe, Harbin and Wuhan cities were summarized in Table 1. Almost all varieties planted in Jinling showed normal maturity before frost except MGVII and MGVIII varieties. Days to maturity of MG000 through MGVI cultivars ranged from 63 to 153 days. However, the very early-maturing varieties showed poor vegetative growth and low yield. The growth duration of MG000 to MG0 reference varieties planted in Heihe was longer than that in Jining (82–115 days vs. 61–80 days). The days to physiological maturity (R7) of MG0 and MGI varieties planted in Harbin were 97–112 days. In Wuhan, late-maturing varieties of MGVI to MGVIII showed a maturity about 142 to 163 days after emergence.

Based on traits of the US soybeans, the maturity standards for the classification of the Chinese soybean MGs at different locations were established (Table 2). As the range of growth duration of varieties within each MG was defined as 10–15 days in the US[1], the classification standard of MG0 and MGI in Harbin could not be used as standard due to a range of less than 10 days. MG000 and MG00 in Jining could not be used as criteria likewise due to the narrow maturity ranges. The references of MGVII and MGVIII could not achieve normal maturity in Jining. Thus, most of the late-maturing soybean failed to be classified specifically in this location, however, normal maturity was achieved in Wuhan city. Although maturity range of the two groups could not meet the standards of 10–15 days in Wuhan, it might also be considered to combine into one late group as a whole which may cover MGVII, MGVIII and even MGIX and MGX. The MG 0 through VI varieties could be used as references in Jining trail, since the maturity met the requirement of 10–15 days (Table 2).

**Table 2:** The Ve-R7 duration ranges of the MG reference varieties from the US in Heihe, Harbin, Jining and Wuhan in China.

MG	Days from emergence (Ve) to physiological maturity (R7)			
	Heihe	Harbin	Jining	Wuhan
000	80–90	—	<71*	—
00	91–101	—		—
0	102–112	101–105	71–81	—
I	—	106–110	82–92	—
II	—	—	93–103	—
III	—	—	104–114	—
IV	—	—	115–125	—
V	—	—	126–138	—
VI	—	—	139–151	141–151
VII/VIII	—	—	≥152*	152–163
MGIX/X	—	—		≥164

* MG000/00MGVII/VIII and MGIX/X were merged respectively at Jining and Wuhan because the groups cannot be distinguished.

### Maturity group classification of Chinese soybean MCC

According to the defined classification criteria (Table 2), the Chinese soybean MCC was classified into different MGs as shown in Table 3. In Heihe trial, 12 varieties grown in spring showed normal maturity before the frost, and were classified into MG000, MG00 and MG0 according to the range of the references. In Wuhan, 57 Chinese MCC soybeans in the trial planted in spring matured normally, most of which were properly classified into different MGs according to the standard of the US references (Table 3). However, the varieties of MGVII and MGVIII could not be distinguished due to similar maturity time. Accessions of MG0-MGIV could be classified based on the growth duration ranges of reference varieties in Jining (Table 2). The classification results of soybeans in Harbin trail showed a consistency of up to 85.7% with that of Jining. Despite the fact that 28 late-maturing accessions could not matured normally in Jining before the frost, and the maturity time was estimated based on the maturity degree of the plants when harvested. The classification of the late-maturing accessions in Jining showed consistency with that of Wuhan, which demonstrated that the data obtained in Jining trial can serve as the main criterion and those from the other three locations can serve as the supplements for the determination of MGs of Chinese soybean MCC.

**Table 3:** Maturity group classification and the genotyping of maturity genes in Chinese soybean MCC.

Collection No.	Varieties	Province	City or countys	Sowing type	MG	R/V	Genotype
ZDD22659	Heifeng37	Heilongjiang	Jiamusi	Spring	0	2.13	E1/E2/e3-1a/E4
ZDD06819	Nenfeng11	Heilongjiang	Nenjiang	Spring	0	1.92	E1/e2-ns/E3/E4
ZDD06822	Hefeng24	Heilongjiang	Jiamusi	Spring	0	1.96	E1/e2-ns/E3/E4
ZDD07623	Jilinchalihua	Jilin	Jilin	Spring	0	-	E1/e2-ns/E3/E4
ZDD03842	Suiyangchunheidoubing	Jiangsu	Shuyang	Spring	0	0.91	E1/e2-ns/E3/E4
ZDD04429	Taixingheidou	Jiangsu	Taixing	Spring	0	1.48	E1/e2-ns/E3/E4
ZDD04430	Taixingaijiaohong	Jiangsu	Taixing	Spring	0	1.61	E1/e2-ns/E3/E4
ZDD06856	Heihexiaohuangdou	Heilongjiang	Heihe	Spring	0	1.76	E1/e2-ns/E3/e4-keshuang
ZDD06851	Dongnong36	Heilongjiang	Harbin	Spring	0	1.02	e1-as/E2/E3/E4
ZDD00326	Fangzhengmoshidou	Heilongjiang	Fangzheng	Spring	0	1.72	e1-as/E2/e3-1a/E4
ZDD17767	Xiaolimoshidou	Heilongjiang	Harbin	Spring	0	1.72	e1-as/E2/e3-Mo/E4
ZDD00046	Kebei1	Heilongjiang	Keshan	Spring	0	1.76	e1-as/e2-ns/E3/E4
ZDD00059	Mufeng1	Heilongjiang	Mudanjiang	Spring	0	1.69	e1-as/e2-ns/E3/E4
ZDD00076	Sinong1	Heilongjiang	Suihua	Spring	0	1.83	e1-as/e2-ns/E3/E4
ZDD22648	Suinong14	Heilongjiang	Suihua	Spring	0	2.26	e1-as/e2-ns/E3/E4
ZDD07409	Hengyoutai	Jilin	Helong	Spring	0	1.63	e1-as/e2-ns/E3/E4
ZDD01421	Liushitianhuancang	Liaoning	Balinyouqi	Spring	0	1.83	e1-as/e2-ns/E3/E4
ZDD00041	Heihe1	Heilongjiang	Heihe	Spring	0	1.75	e1-as/e2-ns/e3-1a/e4-keshuang
ZDD00709	Heimoshidou	Jilin		Spring	I	1.69	E1/E2/e3-1a/E4
ZDD00310	Qinganheidou	Heilongjiang	Qingan	Spring	I	1.91	E1/E2/e3-ns/E4
ZDD06823	Hefeng25	Heilongjiang	Jiamusi	Spring	I	1.83	E1/e2-ns/E3/E4
ZDD07489	Tonghuapingdingxiang	Jilin	Tonghua	Spring	I	1.85	E1/e2-ns/E3/E4
ZDD01629	Baiqidawandou	Hebei	Pingquan	Spring	I	2.22	E1/e2-ns/E3/E4
ZDD18529	Maoyandou	Hebei	Weichang	Spring	I	2.18	E1/e2-ns/E3/E4
ZDD08603	Xiaohuangdou	Shanxi	Huairen	Spring	I	-	E1/e2-ns/E3/E4
ZDD08650	Huangdou<2>	Shanxi	Wuzhai	Spring	I	1.94	E1/e2-ns/E3/E4
ZDD08124	Yanqihuangdou	Xinjiang	Yanqi	Spring	I	2.44	E1/e2-ns/E3/E4
ZDD08125	Changjihuangdou1	Xinjiang	Changji	Spring	I	-	E1/e2-ns/E3/E4
ZDD00698	Chasedou	Jilin	Gongzhuling	Spring	I	1.78	e1-as/E2/E3/E4
ZDD01124	Xiaohuangdou	Liaoning	Chifeng	Spring	I	2.22	e1-as/E2/E3/E4
ZDD00294	Qingdou	Heilongjiang	Acheng	Spring	I	1.95	e1-as/e2-ns/E3/E4
ZDD00603	Changchunmancangjin	Jilin	Changchun	Spring	I	2.17	e1-as/e2-ns/E3/E4
ZDD00638	Bodigao	Jilin	Jiutai	Spring	I	2.13	e1-as/e2-ns/E3/E4
ZDD07218	Zihua2	Jilin		Spring	I	2.28	e1-as/e2-ns/E3/E4
ZDD01074	Xiaobaiqi	Liaoning	Chifeng	Spring	I	1.79	e1-as/e2-ns/E3/E4
ZDD18277	Chi382	Iner Mongolia	Chifeng	Spring	I	1.78	e1-as/e2-ns/E3/E4
ZDD00003	Heinong2	Heilongjiang	Harbin	Spring	I	2.1	e1-as/e2-ns/e3-1a/E4
ZDD11255	77-391-1	Jiangsu	Zhenjiang	Spring	II	2.35	E1/E2/E3/E4
ZDD07088	Longquandadou	Heilongjiang		Spring	II	2.39	E1/e2-ns/E3/E4
ZDD17989	Huangdali	Jilin		Spring	II	2.36	E1/e2-ns/E3/E4
ZDD00854	Jinzhou4-1	Liaoning	Jinzhou	Spring	II	1.87	E1/e2-ns/E3/E4
ZDD00932	Daheiqi	Liaoning	Chaoyang	Spring	II	1.48	E1/e2-ns/E3/E4
ZDD01060	Huangqi	Liaoning		Spring	II	2.55	E1/e2-ns/E3/E4
ZDD01612	Tueryan	Hebei	Pingquan	Spring	II	1.59	E1/e2-ns/E3/E4
ZDD08564	Xiaoyuanhuangdou	Shanxi	Tianzhen	Spring	II	-	E1/e2-ns/E3/E4
ZDD03776	Suiyanchundou	Jiangsu	Huaiyang	Spring	II	1.36	E1/e2-ns/E3/E4
ZDD05494	Honghuliuyuebao	Hubei	Honghu	Spring	II	1.27	E1/e2-ns/E3/E4
ZDD05502	Nidou	Hubei	Wuchang	Spring	II	1.29	E1/e2-ns/E3/E4
ZDD11575	Huasedou	Hubei	Chongyang	Spring	II	1.3	E1/e2-ns/E3/E4
ZDD06515	Xiangdou4	Hunan	Changsha	Spring	II	1.23	E1/e2-ns/E3/E4
ZDD20671	Gongdou7	Sichuan	Zigong	Spring	II	1.67	E1/e2-ns/E3/E4
ZDD14240	Duchangwudou	Jiangxi	Duchang	Spring	II	1.54	E1/e2-ns/E3/E4
ZDD06454	Hengfengwudou	Jiangxi	Hengfeng	Spring	II	1.49	E1/e2-ns/E3/E4
ZDD14252	Fengchengzaowudou	Jiangxi	Fengcheng	Spring	II	1.42	E1/e2-ns/E3/E4
ZDD22207	Madaiheidou-3	Guangdong	Lianxian	Spring	II	1.38	E1/e2-ns/E3/E4
ZDD03026	Pingdinghei	Shandong	Taian	Summer	II	1.49	E1/e2-ns/E3/E4
ZDD18632	Jidou7	Hebei	Shijiazhuang	Summer	II	2.31	e1-as/e2-ns/E3/E4
ZDD08190	Yangtianxiaohuangdou	Hebei	Chicheng	Spring	III	1.77	E1/E2/E3/E4
ZDD08228	Nanguanxiaopiqing	Hebei	Qianxi	Spring	III	1.57	E1/E2/E3/E4
ZDD18524	Xiaotaizimoshidou	Hebei	Xinglong	Spring	III	1.66	E1/E2/E3/E4
ZDD08018	Miuyunlaoyelian	Beijing	Miyun	Spring	III	1.57	E1/E2/E3/E4
ZDD08472	Heidou	Hebei	Wuyi	Summer	III	1.35	E1/E2/E3/E4
ZDD03106	Chadou	Shandong	Qihe	Summer	III	1.55	E1/E2/E3/E4
ZDD01402	Daheilidou	Liaoning	Balinyouqi	Spring	III	3.23	E1/e2-ns/E3/E4
ZDD01489	Yushidou	Liaoning	Xingcheng	Spring	III	1.52	E1/e2-ns/E3/E4
ZDD02096	Tianedan	Shanxi	Wuxiang	Spring	III	2.23	E1/e2-ns/E3/E4
ZDD02159	Daheidou	Shanxi	Daixian	Spring	III	2.00	E1/e2-ns/E3/E4
ZDD08928	Liushiribaidou	Shanxi	Yicheng	Spring	III	1.61	E1/e2-ns/E3/E4
ZDD10186	Zaoshuhuangdou	Shaanxi	Taibai	Spring	III	1.74	E1/e2-ns/E3/E4
ZDD03739	Pixiandazihuacao	Jiangsu	Pixian	Spring	III	2.18	E1/e2-ns/E3/E4
ZDD03740	Pixiannianzhuangliuyuexian	Jiangsu	Pixian	Spring	III	1.59	E1/e2-ns/E3/E4
ZDD14505	Yizhangliuyuehuang	Hunan	Yizhang	Spring	III	1.18	E1/e2-ns/E3/E4
ZDD12436	Bazhongtiankandou	Sichuan	Bazhong	Spring	III	1.25	E1/e2-ns/E3/E4
ZDD12527	Pixiangxiaohuangdou	Sichuan	Pixian	Spring	III	1.30	E1/e2-ns/E3/E4
ZDD20676	Liuyuehuang	Sichuan	Deyang	Spring	III	1.29	E1/e2-ns/E3/E4
ZDD21030	Pengshanghuangkezi-3	Sichuan	Pengshan	Spring	III	1.36	E1/e2-ns/E3/E4
ZDD15357	Dahuangdou-1	Guzhou	Zunyi	Spring	III	1.38	E1/e2-ns/E3/E4
ZDD14228	Wuyuehuang	Jiangxi	Yongxin	Spring	III	1.48	E1/e2-ns/E3/E4
ZDD06363	Dalihuang	Fujian	Jinjiang	Spring	III	1.58	E1/e2-ns/E3/E4
ZDD06377	Xiamentengzidou	Fujian	Xiamen	Spring	III	1.26	E1/e2-ns/E3/E4
ZDD06378	Tonganzihongdou	Fujian	Tongan	Spring	III	1.21	E1/e2-ns/E3/E4
ZDD16675	Dabaimaodou	Guangdong	Heping	Spring	III	1.23	E1/e2-ns/E3/E4
ZDD18835	Maoyandou	Hebei	Weixian	Summer	III	2.43	E1/e2-ns/E3/E4
ZDD01720	Sijiaoqihuangdou	Hebei	Quzhou	Summer	III	1.36	E1/e2-ns/E3/E4
ZDD08352	Bendidahuangdou	Hebei	Xianxian	Summer	III	1.33	E1/e2-ns/E3/E4
ZDD18771	Qingdou	Hebei	Lingshou	Summer	III	1.41	E1/e2-ns/E3/E4
ZDD02626	Shengli3	Shandong	Yiyuan	Summer	III	1.93	E1/e2-ns/E3/E4
ZDD02940	Lücaodou	Shandong	Mengyin	Summer	III	1.39	E1/e2-ns/E3/E4
ZDD19409	Zheng84240-B1	Henan	Zhengzhou	Summer	III	1.67	E1/e2-ns/E3/E4
ZDD03570	Xinayngyangyandou	Henan	Xinyang	Summer	III	1.43	E1/e2-ns/E3/E4
ZDD03868	Peixianxiaoyoudou	Jiangsu	Peixian	Summer	III	1.20	E1/e2-ns/E3/E4
ZDD04959	ZDD04959	Anhui	Wanbei	Summer	III	1.79	E1/e2-ns/E3/E4
ZDD12845	Jiangehualinjiwodou	Sichuan	Jiange	Summer	III	1.27	E1/e2-ns/E3/E4
ZDD12908	Qionglaiyoujiangheidou	Sichuan	Qionglai	Summer	III	1.21	E1/e2-ns/E3/E4
ZDD01169	Niumaohuang	Liaoning	Gaixian	Spring	III	1.51	e1-as/E2/E3/E4
ZDD08238	Chichenglvhuangdou	Hebei	Chicheng	Spring	III	2.33	e1-as/E2/E3/E4
ZDD08690	Xiaohuandou	Shanxi	Yuci	Spring	III	2.47	e1-as/E2/E3/E4
ZDD19381	Gaozuoxuan1	Shandong	Gaomi	Summer	III	2.18	e1-as/E2/E3/E4
ZDD01983	Baipihuangdou	Shanxi	Qiuling	Spring	IV	2.53	E1/E2/E3/E4
ZDD04275	Tongshanqingdadou	Jiangsu	Tongshan	Summer	IV	1.38	E1/E2/E3/E4
ZDD04620	Taixingniumaohuangyi	Jiangsu	Taixing	Summer	IV	1.24	E1/E2/E3/E4
ZDD13666	Lülanzi	Sichuan	Xichang	Summer	IV	1.18	E1/E2/E3/E4
ZDD02134	Xiaohuangdou	Shanxi	Lingchuan	Spring	IV	2.46	E1/e2-ns/E3/E4
ZDD08728	Bailudou	Shanxi	Heshun	Spring	IV	2.58	E1/e2-ns/E3/E4
ZDD03728	Suiningpingdinghuang	Jiangsu	Suining	Spring	IV	-	E1/e2-ns/E3/E4
ZDD12331	Xiaobaimao	Sichuan	Pixian	Spring	IV	1.4	E1/e2-ns/E3/E4
ZDD12635	Zizhongliuyuezao	Sichuan	Zizhong	Spring	IV	1.48	E1/e2-ns/E3/E4
ZDD12680	Jianweiquanshuidou	Sichuan	Jianwei	Spring	IV	1.22	E1/e2-ns/E3/E4
ZDD20652	8307/8/1	Sichuan	Chengdu	Spring	IV	1.53	E1/e2-ns/E3/E4
ZDD14920	Erjizaozou-2	Guzhou	Zhijin	Spring	IV	1.2	E1/e2-ns/E3/E4
ZDD15624	Zaojiaodou	Guzhou	Xiuwen	Spring	IV	1.35	E1/e2-ns/E3/E4
ZDD06375	Daqingren	Fujian	Jinjiang	Spring	IV	1.46	E1/e2-ns/E3/E4
ZDD14125	Pudou451	Fujian	Putian	Spring	IV	1.37	E1/e2-ns/E3/E4
ZDD21485	Quanbian11	Fujian	Quanzhou	Spring	IV	1.47	E1/e2-ns/E3/E4
ZDD06358	Dongshanbaimadou	Fujian	Dongshan	Spring	IV	1.55	E1/e2-ns/E3/E4
ZDD01683	Diliuhuangsou-2	Hebei	Gaocheng	Summer	IV	1.45	E1/e2-ns/E3/E4
ZDD18558	Huaheihu	Hebei	Hejian	Summer	IV	1.42	E1/e2-ns/E3/E4
ZDD02764	Siliyuan	Shandong	Zaozhuang	Summer	IV	1.45	E1/e2-ns/E3/E4
ZDD02866	Dabaipi	Shandong	Weishan	Summer	IV	1.72	E1/e2-ns/E3/E4
ZDD02913	Xiaomidou	Shandong	Dongping	Summer	IV	1.53	E1/e2-ns/E3/E4
ZDD19131	Maodou	Shandong	Zhaoyuan	Summer	IV	2.12	E1/e2-ns/E3/E4
ZDD10100	Zheng8516	Henan	Zhengzhou	Summer	IV	1.58	E1/e2-ns/E3/E4
ZDD04918	ZDD04918	Anhui	Wanbei	Summer	IV	1.71	E1/e2-ns/E3/E4
ZDD12023	Chihuangdou1	Hubei	Lichuan	Summer	IV	1.10	E1/e2-ns/E3/E4
ZDD12872	Qionglaihuangmaozi	Sichuan	Qionglai	Summer	IV	1.29	E1/e2-ns/E3/E4
ZDD17325	Xuanza	Yunnan	Xuanwei	Summer	IV	2.10	E1/e2-ns/E3/E4
ZDD22145	Dahuangdou-2	Guangdong	Jiaoling	Spring	IV	1.29	E1/e2-ns/e3-tr/E4
ZDD08633	Qingkeyuandou	Shanxi	Daixian	Spring	IV	2.01	e1-as/E2/E3/E4
ZDD19699	Sidou2	Jiangsu	Siyang	Summer	IV	2.16	e1-as/E2/E3/E4
ZDD02400	Xiaoheidou	Shanxi	Wuxiang	Spring	V	1.91	E1/E2/E3/E4
ZDD09279	Xiaoheidou	Shanxi	Yixian	Spring	V	1.79	E1/E2/E3/E4
ZDD19027	Lüpihuangdou	Shanxi	Gujiao	Spring	V	2.73	E1/E2/E3/E4
ZDD10252	Xiaoheidou	Shaanxi	Fugu	Spring	V	1.52	E1/E2/E3/E4
ZDD10270	Xiaoheidou	Shaanxi	Dingbian	Spring	V	2.15	E1/E2/E3/E4
ZDD08120	Nidinghuameidou	Ningxia	Zhongning	Spring	V	1.81	E1/E2/E3/E4
ZDD14911	Xihuangdou-9	Guzhou	Zhijin	Spring	V	0.94	E1/E2/E3/E4
ZDD16743	Lianjiangpohuangdou	Guangdong	Lianjiang	Spring	V	0.88	E1/E2/E3/E4
ZDD19293	Zaoshuheidou	Shandong	Rushan	Summer	V	3.07	E1/E2/E3/E4
ZDD03540	Boaihongpizaojiaozi	Henan	Boai	Summer	V	2.74	E1/E2/E3/E4
ZDD11586	82–16	Hubei	Wuhan	Summer	V	1.16	E1/E2/E3/E4
ZDD17457	Yangyandou	Yunnan	Yongde	Summer	V	1.31	E1/E2/E3/E4
ZDD11092	Youhuangdou	Gansu	Xihe	Spring	V	2.27	E1/E2/E3/E4
ZDD00921	Tianedan	Liaoning	Jinzhou	Spring	V	2.12	E1/e2-ns/E3/E4
ZDD02114	Tianedan	Shanxi	Tunliu	Spring	V	2.92	E1/e2-ns/E3/E4
ZDD08697	Yuxuan13	Shanxi	Yuci	Spring	V	2.39	E1/e2-ns/E3/E4
ZDD09136	Xiaoqingdou	Shanxi	Hongdong	Spring	V	2.08	E1/e2-ns/E3/E4
ZDD10359	Laoheidou	Shaanxi	Zihou	Spring	V	2.56	E1/e2-ns/E3/E4
ZDD03733	Pixianhongmaoyou	Jiangsu	Pixian	Spring	V	1.90	E1/e2-ns/E3/E4
ZDD03741	Pixiansiyuecao	Jiangsu	Pixian	Spring	V	1.39	E1/e2-ns/E3/E4
ZDD06562	Baimaodou	Guzhou	Xingyi	Spring	V	1.5	E1/e2-ns/E3/E4
ZDD10430	Huichaxiaohuangdou	Shaanxi	Luonan	Summer	V	1.55	E1/e2-ns/E3/E4
ZDD10812	Jianghuangdou	Shaanxi	Lantian	Summer	V	1.7	E1/e2-ns/E3/E4
ZDD02864	Pingdianghuangdou	Shandong	Weishan	Summer	V	1.86	E1/e2-ns/E3/E4
ZDD02891	Dahuangdou	Shandong	Liangshan	Summer	V	1.82	E1/e2-ns/E3/E4
ZDD02892	Datianedan	Shandong	Liangshan	Summer	V	1.84	E1/e2-ns/E3/E4
ZDD02921	Qing6	Shandong	Taian	Summer	V	1.97	E1/e2-ns/E3/E4
ZDD19144	Qisiwa	Shandong	Wendeng	Summer	V	1.93	E1/e2-ns/E3/E4
ZDD03153	Miyangxiaozihuang	Henan	Miyang	Summer	V	1.39	E1/e2-ns/E3/E4
ZDD03237	Xichuanjiwohuang	Henan	Xichuan	Summer	V	1.53	E1/e2-ns/E3/E4
ZDD03293	Miyangniumaohuang	Henan	Miyang	Summer	V	1.18	E1/e2-ns/E3/E4
ZDD03533	Zhechengxiaohongdou	Henan	Zhecheng	Summer	V	2.01	E1/e2-ns/E3/E4
ZDD11581	82–24	Hubei	Wuhan	Summer	V	1.46	E1/e2-ns/E3/E4
ZDD11588	74–424	Hubei	Wuhan	Summer	V	1.56	E1/e2-ns/E3/E4
ZDD11703	Shuguanghuangdou	Hubei	Dangyang	Summer	V	1.12	E1/e2-ns/E3/E4
ZDD12386	Dahuangdou	Sichuan	Dayi	Summer	V	1.18	E1/e2-ns/E3/E4
ZDD13560	Baimaozaodouzi	Sichuan	Ningnan	Summer	V	0.87	E1/e2-ns/E3/E4
ZDD17622	Malanzaochadou	Yunnan	Zhaotong	Summer	V	2.08	E1/e2-ns/E3/E4
ZDD18870	Dongshan69	Shanxi	Taiyuan	Spring	V	2.92	e1-as/E2/E3/E4
ZDD02315	Huipizhiheidou	Shanxi	Xingxian	Spring	VI	1.47	E1/E2/E3/E4
ZDD16954	Bozhidou	Guangxi	Lingshan	Spring	VI	0.60	E1/E2/E3/E4
ZDD16771	Qingyuandaqingdou	Guangdong	Qingyuan	Spring	VI	0.71	E1/E2/E3/E4
ZDD04572	Wujiangwuyueniumaohuang	Jiangsu	Wujiang	Summer	VI	1.49	E1/E2/E3/E4
ZDD04604	Yizhengdalihuangdou	Jiangsu	Yizheng	Summer	VI	0.84	E1/E2/E3/E4
ZDD11323	Dantuxiaowujia	Jiangsu	Dantu	Summer	VI	0.69	E1/E2/E3/E4
ZDD17375	Huangdou	Yunnan	Yongli	Summer	VI	1.43	E1/E2/E3/E4
ZDD21907	Xinyudaliqing	Jiangxi	Xinyu	Autumn	VI	1.49	E1/E2/E3/E4
ZDD14190	Baiqiu1	Fujian	Sanming	Autumn	VI	-	E1/E2/E3/E4
ZDD08986	Xiaobaidou<2>	Shanxi	Wenxi	Spring	VI	1.11	E1/e2-ns/E3/E4
ZDD12828	Suiningtaisejianghuangdou	Sichuan	Suining	Spring	VI	1.40	E1/e2-ns/E3/E4
ZDD03603	Niumaohuang	Shaanxi	Zhenan	Summer	VI	1.29	E1/e2-ns/E3/E4
ZDD11159	Hualvhuangdou	Gansu	Liangdang	Summer	VI	1.26	E1/e2-ns/E3/E4
ZDD03969	Pixianlayanghuang	Jiangsu	Pixian	Summer	VI	1.07	E1/e2-ns/E3/E4
ZDD04092	Binhaidahuangkezijia	Jiangsu	Binhai	Summer	VI	1.49	E1/e2-ns/E3/E4
ZDD11226	Guanyunhaibaihua	Jiangsu	Guanyun	Summer	VI	1.08	E1/e2-ns/E3/E4
ZDD05920	Daimidou	Hubei		Summer	VI	1.10	E1/e2-ns/E3/E4
ZDD11624	Chihuangdou2	Hubei	Luotian	Summer	VI	0.93	E1/e2-ns/E3/E4
ZDD11951	Shanzibaihuangdou	Hubei	Yunxi	Summer	VI	1.15	E1/e2-ns/E3/E4
ZDD13401	Liuyuebao-2	Sichuan	Yaan	Summer	VI	1.87	E1/e2-ns/E3/E4
ZDD19579	Lühuangdou	Gansu	Chongxin	Spring	VII/VIII	2.06	E1/E2/E3/E4
ZDD16756	Yangshanqingdou	Guangdong	Yangshan	Spring	VII/VIII	0.79	E1/E2/E3/E4
ZDD20340	Lürouheipidou	Anhui	Yuexi	Summer	VII/VIII	1.64	E1/E2/E3/E4
ZDD12322	Huameidou	Hubei	Wufeng	Summer	VII/VIII	1.02	E1/E2/E3/E4
ZDD20532	Xiaolihuangdou	Hubei	Zhuxi	Summer	VII/VIII	0.84	E1/E2/E3/E4
ZDD12389	Wuyanwo	Sichuan	Beichuan	Summer	VII/VIII	1.4	E1/E2/E3/E4
ZDD12407	Zengjialvhuangdou	Sichuan	Guangyuan	Summer	VII/VIII	1.31	E1/E2/E3/E4
ZDD12415	Mayibao	Sichuan	Yunyang	Summer	VII/VIII	-	E1/E2/E3/E4
ZDD06475	Yantiaoqingpidou	Jiangxi	Wuyuan	Summer	VII/VIII	-	E1/E2/E3/E4
ZDD06067	Cudou	Zhejiang	Pinghu	Summer	VII/VIII	0.60	E1/E2/E3/E4
ZDD06501	Ruijinqingpidou	Jiangxi	Ruijin	Autumn	VII/VIII	-	E1/E2/E3/E4
ZDD21440	Zaoshumaopengqing	Zhejiang	Quzhou	Autumn	VII/VIII	1.24	E1/E2/E3/E4
ZDD14409	Dahuangzhu	Jiangxi	Qianshan	Autumn	VII/VIII	0.61	E1/E2/E3/E4
ZDD10539	Dahuangdou	Shaanxi	Zhenba	Summer	VII/VIII	1.45	E1/e2-ns/E3/E4
ZDD13636	Lvdouzi	Sichuan	Mingshan	Summer	VII/VIII	1.19	E1/e2-ns/E3/E4
ZDD13341	Qiyuehuang	Sichuan	Mianyang	Summer	VII VIII		E1/E2/E3/E4
ZDD16846	Yingdehedou	Guangdong	Yingde	Spring	VII/ VIII	0.71	E1/E2/E3/E4
ZDD19464	Baomuji	Shaanxi	Zhenan	Summer	VII/ VIII	-	E1/E2/E3/E4
ZDD10572	Niupihuangdou	Shaanxi	Pingli	Summer	VII/ VIII	-	E1/E2/E3/E4
ZDD05572	Jinghuang35yi	Hubei		Summer	VII/ VIII	0.68	E1/E2/E3/E4
ZDD11866	Chahuangdaidou	Hubei	Nanzhang	Summer	VII/ VIII	0.88	E1/E2/E3/E4
ZDD17233	Mashanrenfenghuangdou	Guangxi	Mashan	Summer	VII/ VIII	-	E1/E2/E3/E4
ZDD16874	Heikewudou	Hainan	Chengmai	Summer	VII/ VIII	-	E1/E2/E3/E4
ZDD12836	Shifangluosidou	Sichuan	Shifang	Spring	VII/ VIII	1.39	E1/e2-ns/E3/E4
ZDD10615	Laoshupi	Shaanxi	Ningshan	Summer	VII/ VIII	1.18	E1/e2-ns/E3/E4
ZDD17574	Songzidou	Yunnan	Huaning	Summer	VII/ VIII	2.00	E1/e2-ns/E3/E4
ZDD12688	Changshouxhiyuehuang	Sichuan	Changshou	Spring	IX/X	-	E1/E2/E3/E4
ZDD12910	Hanyuanbalixiaoheidou	Sichuan	Hanyuan	Summer	IX/X	-	E1/E2/E3/E4
ZDD13233	Donghuangdou1	Sichuan	Shizhu	Summer	IX/X	-	E1/E2/E3/E4
ZDD13441	Zaohuangdou-4	Sichuan	Xingjing	Summer	IX/X	-	E1/E2/E3/E4
ZDD12400	Shiyuehuang	Sichuan	Xichang	Summer	IX/X	-	E1/E2/E3/E4
ZDD06461	Shangraobayuebai	Jiangxi	Shangrao	Summer	IX/X	-	E1/E2/E3/E4
ZDD06803	Dawudo	Guangxi	Hepu	Summer	IX/X	-	E1/E2/E3/E4
ZDD06528	Huangmaodou	Hunan	Ningyuan	Autumn	IX/X	-	E1/E2/E3/E4
ZDD06543	Hongzhudou	Hunan	Hengshan	Autumn	IX/X	-	E1/E2/E3/E4
ZDD14782	Changshanidou	Hunan	Changsha	Autumn	IX/X	-	E1/E2/E3/E4
ZDD14783	Aishengnidou	Hunan	Liuyang	Autumn	IX/X	-	E1/E2/E3/E4
ZDD06494	Shaxindou	Jiangxi	Shicheng	Autumn	IX/X	-	E1/E2/E3/E4
ZDD06410	Zhaoanqiudadou	Fujian	Zhaoan	Autumn	IX/X	-	E1/E2/E3/E4
ZDD06438	Shaxianwudou	Fujian	Shaxian	Autumn	IX/X	-	E1/E2/E3/E4

### Distribution of Chinese soybean MCC in geographic regions and maturity groups

The Chinese soybean MCC and the MG assignments in the provinces were listed in Tables 4 and 5, respectively. MCC covered a large range of MGs from MG000 to MGIX/X. Spring-sowing soybeans were primarily distributed in MG 000 through MGV, and a few of which from south China were in late-maturing groups with rich genetic basis of maturity in the spring-sowing varieties. Soybeans of summer-sowing type were mainly distributed in the MG II through MGIX/X, whereas the autumn-sowing varieties were sorted into MGVI or later. This indicated the MG range of these accessions was narrow. MG000 and MG00 MCC were only distributed in Northern Heilongjiang, and MGIX and MGX were only found in the accessions from south China. MGIII contained the largest number of accessions which were collected from different regions. Fifty-five accessions in MGIII originated from 16 provinces were mainly spring-sowing or summer-sowing types. Soybeans of MGVII/VIII included spring- summer- and autumn-sowing types. Soybean cultivars in Sichuan Province showed the largest number of 30 accessions, while those of the Heilongjiang, Shanxi, Shandong and Jiangsu provinces were more than 20 accessions. However, there was no MCC accession from Tibet Autonomous Region, Qinghai province, and Tianjin, Shanghai and Chongqing Municipalities, respectively.

**Table 4:** Maturity group assignments of the Chinese soybean MCC.

MG	Variety
000	**Spring-sowing**: Dongnong36, WilenskabaranatraII-2-184, Pojabonar 856–3, Heihexiaohuangdou
00	**Spring-sowing:** Heihe1, Hefeng37
0	**Spring-sowing:** mufeng1, hefeng24, helongyoutai, shuyangchunheidoubing, baichengmoshidou, Boige du lot et geronne, fangzhengmoshidou, dongnong434, kebei1, liushitianhuancang, suinong6, suinong1, Dunajka, taixingaijiaohong, jilinchalihua, zhengguang1, Flora, xiaolimoshidou, nenfeng11, taixingheidou, suinong14, dongnong163
I	**Spring-sowing:** hefeng25, huangdou<2>, chasedou, qingdou, yanqihuangdou, yapoche, chi382, qinganheidou, zihua2, changchunmancangjin, maoyandou(Jinlin), xiaohuangdou, heinong2, bodigao, xiaobaiqi, heimoshidou, tonghuapingdingxiang, Icar 166, jinshanchamoshidou, xiaohuangdou, maoyandou(Liaoning), baiqidawandou, datunxiaoheidou, jinlin30, changjihuangdou1
II	**1. Spring-sowing:** nidou, hengfengwudou, jinzhou4-1, 60 CMS superspecial, 77-391-1, duchangwudou, zhechun2, Domaka tolisa, huangqi, xiaoyuanhuangdou, hongfeng11, gongdou7, xiangdou4, longquandadou, Harosoy, daheiqi, tueryan, zhechun3, Harosoy2, huasedou, fengchengzaowudou, huangdali, honghuliuyuebao, huaiyangchundou, madaiheidou-3 **2. Summer-sowing:** jidou12, jidou7, pingdinghei, ludou4, erliheidou, zaoshu18, zhongdou27
III	**1. Spring-sowing**: Nova, wuyuehuang, bazhongtiankandou, tianedan, pixiannianzhuangliuyuexian, dabaimaodou, pixiandazihuacao, xiaohuangdou(Shanxi), xiamentengzidou, longchuanhuangniumao, daliheidou, yizhangliuyuehuang, xiaohuangdou(Liaoning), xiataiximoshidou, damingbaidouzi<2>, tonganzihongdou, fengjiao66-22, pixianxiaohuangdou, liuyuehuang, panshidou, liushiribaidou, chichenglyuhuangdou, niumaohuang, nanguanxiaopiqing, yushidou, Williams, tianedan, dahuangdou-1, yangtianxiaohuangdou, dalihuang, miyunlaoyelian, pengshanhuangkezi-3, zaoshuhuangdou, daheidou **2. Summer-sowing:** chadou, bendidahuangdou, jingehualinjiwodou, zhonghuang4, lyucaodou, yanhuang3, qionglaiyoujiangheidou, zheng92116, zheng84240-B1, heidou, peixianxiaoyoudou, xinyangyangyandou, sijiaoqihuangdou, shengli3, yudou27, puhai10, ZDD04959, gaozuoxuan1, maoyandou(Hebei), qingdou(Hebei), Clark
IV	**1. Spring-sowing**: dongshanbaimadou, pudou451, zizhongliuyuezao, dongguanwuyuehuang, chamoshidou, dahuangdou-2(Guangdong), zaojiaodou, erjizaodou-2, daqingren, 8307-8-1, Shinpaldal kong2, baipihuangdou, jianweiquanshuidou, qingkeyuandou, xiaobaimao, PI486355,gui199, suiningpingdinghuang, pixiandasanjiaodou, xiaohuangdou(Shanxi), lyugundou, bailudou, quanbian11 **2. Summer-sowing:** qionglaihuangmaozi, lyulanzi, huaheihu, liuliuhuangdou-2, zheng8516, tongshnqingdadou, xuanza, chihuangdou1, suqiandadudou, ZDD04918, taixingniumaohuangyi, sidou2, 7651–1, wenfeng7, maodou, maodou, yuandou, siliyuan, xiaomidou, dabaipi
V	**1. Spring-sowing:** pixianhongmaoyou, xiaoheidou, pixiansilicao, xiaoqingdou, nidinghuameidou, tianedan, cansidou, Hartwig, tianedan(Shanxi), dongshan69, lianjiangpohuangdou, heheidou, xihuangdou-9, xiaoheidou(Shaan xi), baimaodou, lyupihuangdou, youhuangdou, xiaheidou, yuxuan10, laoheidou **2. Summer-sowing:** dahuangdou, malanzaochadou, boaihongpizaojiaozi, xichuanjiwohuang, datianedan, 5081, zhongte1, shuguanghuangdou, pingdinghuangdou, qisiwa, zaoshuheidou, dazhonghua, qing6, chadou(Shandong), zhechengxiaohongdou, 82–24, jinghuangdou, 74–424, 82–16, huichaxiaohuangdou, yangyandou, dalyudou, miyangxiaozihuang, baimaozaodouzi, miyangniumaohuang
VI	**1. Spring-sowing:** qingdou(Shanxi), xiaobaidou, huipizhiheidou, suiningtaifengjiangsedou, baizhidou, qingyuandaqingdou **2. Summer-sowing:** heidou(Shaanxi), wujiangwuyueniumaohuang, pixianlayanghuang, binhaidahuangkezijia, niumaohuang, hualyuhuangdou, binhaiwuhuazuanding, guanyunhaibaihua, liuyuebao-2, shanzibaihuangdou, lyupidou, chihuangdou2, huangdou(Yunnan), daimidou, yizhengdalihuangdou, dantuxiaohuangjia **3. Autumn-sowing:** Baiqiu1, Xinyudaliqing
VII/VIII	**1. Spring-sowing:** yingdeihedou, shifangluosidou, lyuhuangdou, yangshanqingdou **2. Summer-sowing:** lyudouzi, jinghuang35, wuyanwo, mochadaidou1, dahuangdou(Shaanxi), lyurouheipidou, yantianqingpidou, laoshupi, zengjialyuhuangdou, niupihuangdou, qingdou(Zhejiang), baomuji, AGS190, xiaokehuangdou, huameidou, cudou, songzidou, heikewudou, mayibao, qiyuehuang, mashanrenfenghuangdou **3. Autumn-sowing**: ruijinqingpidou, dahuangzhu, ITAL SOJA-2, zaoshumaopengqing
MGIX/X	**1. Spring-sowing:** Changshoushiyuehuang **2. Summer-sowing:** shangraobayuebai, zaohuangdou-4, huanyuanbalixiaoheidou, Nigeria5, shiyuehuang, donghuangdou-1, dawudou, diaolianbao-2, baishuidou, lyu75 **3. Autumn-sowing:** shaxindou, changshanidou, aishengnidou, hongzhudou, huangmaodou, zhaoanqiudadou, shaxianwudou

**Table 5:** Maturity group assignment of Chinese soybean MCC in certain provinces in China.

Origin	Sowing type	Number of accessions	Number of accessions										
			000	00	0	I	II	III	IV	V	VI	VII/VIII	IX/X
Heilongjiang	Sp	22	2	2	11	5	2						
Jilin	Sp	15			4	8	1	1	1				
Liaoning	Sp	14			1	3	3	6		1			
Inner Mongolia	Sp	1				1							
Xinjiang	Sp	2				2							
Ningxia	Sp	1								1			
Gansu	Sp, Su	4							1	1	1	1	
Shaanxi	Sp, Su	13						1		5	3	4	
Shanxi	Sp	24				2	1	5	4	9	3		
Hebei	Sp, Su	19				3	3	9	4				
Beijing	Sp, Su	3					1	2					
Shandong	Su	20					3	5	5	7			
Henan	Su	11						5	1	5			
Jiangsu	Sp, Su	22			3		2	3	5	2	7		
Anhui	Su	4						1	1	1		1	
Hubei	Sp, Su	16					4		1	4	3	4	
Hunan	Sp, Au	6					1	1					4
Jiangxi	Sp, Su, Au	10					3	1			1	3	2
Sichuan	Sp, Su	30					1	6	7	2	2	6	6
Guizhou	Sp, Su	6						1	2	2			1
Yunnan	Su	6							1	3	1	1	
Zhejiang	Sp, Su, Au	4					1					3	
Fujian	Sp, Au	10						3	4		1		2
Guangdong	Sp	9					1	2	2	1	1	2	
Guangxi	Sp, Su	4							1		1	1	1
Hainan	Su	1										1	
North America	Sp	22	2		3	1	5	3	2	2		2	2
Total		299	4	2	22	25	32	55	42	46	24	29	18

Twenty MCC accessions originated from 16 foreign countries also showed large range from MG000 to MGIX/X except the MGVI. All the 15 spring-sowing soybean accessions were allocated from MG 000 to MGVI, and soybeans of summer- and autumn-sowing types were mainly distributed into the groups above MGV. MGII was the largest group which included more foreign varieties than other MGs.

### Growth period structure (R/V) variation of Chinese soybean MCC

The R/V ratio of 244 soybean varieties was calculated using the data obtained from Jining under spring-sowing conditions. There were wide variation in the ratio of reproductive (R) to vegetative (V) periods (R/V) among the MCC ranging from 0.60 to 3.23 (Table 3). The summer-sowing variety of Cudou from Zhejiang province classified into MGVII/VIII showed the minimum ratio (0.60) while the spring-sowing variety of Daliheidou of MGIII from Jilin showed the maximum value (3.23). The varieties of the same MG also showed a remarkable variation in the R/V ratio. Among the MGIII varieties, the maximum R/V ratio was up to 2.63 (Williams), but the minimum value was only 1.18 (Yizhangsiyuehuang). Among the MGI accessions, the maximum R/V ratio (Yanqihuangdou) and the minimum value (Datunxiaoheidou) was 2.44 and 1.51, respectively. Varieties from the same region were also diverse in the R/V ratio. For example, R/V ratio of varieties from Sichuan province ranged from 0.87 to 1.87. Compared with Chinese accessions, the foreign germplasm in the MCC also showed diversity in the R/V ratio. Significant variation of the R/V ratio which was observed among the varieties reflected the rich genetic background of MCC.

### Genotyping of the maturity genes in Chinese soybean MCC

In order to detect alleles of the maturity genes in Chinese soybean MCC, 228 soybean accessions (76.3%) were genotyped by the Sequenom MassARRAY platform at four maturity loci (i.e. *E1-E4*) and a total of twelve genotypes were identified in this population. Among all these groups, genotypes of *E1/e2-ns/E3/E4* and E1/E2/E3/E4 were the major types, which were identified in 128 and 68 cultivars, respectively (Table 3). Seven genotypes, including *E1/E2/e3-ns/E4*, *E1/e2-ns/E3/e4-keshuang*, *E1/e2-ns/e3-tr/E4*, *e1-as/E2/e3-la/E4*, *e1-as/E2/e3-Mo/E4*, *e1-as/e2-ns/e3-la/E4*, *e1-as/e2-ns/e3-la/e4-keshuang*, were identified only in one variety (Table 3). In addition, the other 3 genotypes (e.g. *E1/E2/e3-1a/E4*, *e1-as/E2/E3/E4*, *e1-as/e2-ns/E3/E4*) were identified in 2, 10, and 13 accessions, respectively.

To determine the effects of maturity genes on maturity and photoperiod response in MCC, the relationship between allelic constitutions and maturity groups of each kind of genotypes were analyzed (Table 6). The results showed that *E1/E2/E3/E4* genotypes were always detected in medium and late-maturing accessions from MGII to VIII and even MGIX/X. In contrast, the recessive allele of the *E3* and *E4* was always detected in varieties from MG0 and MGI except a variety Dahuangdou-2 belonging to MGIV with two recessive alleles in *E2* and *E3* loci originated from Guangdong province, southern coast area of China. The allele *e4* was detected in only two cultivars belonging to MG0, both of which were from Heihe, Heilongjiang Province with high latitude and low temperature[35].

**Table 6:** The distribution of allelic variation of *E1*, *E2*, *E3* and *E4* loci in different MGs of Chinese soybean MCC.

MGs	*Genotypes*											
	*G1*	*G2*	*G3*	*G4*	*G5*	*G6*	*G7*	*G8*	*G9*	*G10*	*G11*	*G12*
0		1		6	1		1	1	1	6		1
I		1	1	8			2			6	1	
II	1			18						1		
III	6			31			4					
IV	4			24		1	2					
V	13			25			1					
VI	9			11								
VII/VIII	21			5								
IX/X	14											

*Note*: *G1*: *E1/E2/E3/E4*; *G2*: *E1/E2/e3-1a/E4*; *G3*: *E1/E2/e3-ns/E4*; *G4*: *E1/e2-ns/E3/E4*; *G5*: *E1/e2-ns/E3/e4-keshuang*; *G6*: *E1/e2-ns/e3-tr/E4*; *G7*: *e1-as/E2/E3/E4*; *G8*: *e1-as/E2/e3-1a/E4*; *G9*: *e1-as/E2/e3-Mo/E4*; *G10*: *e1-as/e2-ns/E3/E4*; *G11*: *e1-as/e2-ns/e3-1a/E4*; *G12*: *e1-as/e2-ns/e3-1a/e4-keshuang*.

## Discussion

MCC is a small sub-set of the entire collection which represents most of the total genetic variation with a minimal redundancy[53](Brown, 1995). Therefore, it is an ideal choice to utilize the MCC of soybean in China as representative materials to investigate the maturity diversity among Chinese varieties[5]. As shown in Table 5, accessions of MCC originated from 26 provinces and other 16 countries spanned more than 12 MGs (MG000 to MGIX/X). Moreover, rare alleles of maturity genes were also identified in this population, despite some genotypes were only detected in a single accession. These results were consistent with a previous report about the MG scope of Chinese soybean[54], which confirmed the representation and high diversity of this collection.

Gai et al. (2001) and Wang et al. (2006) suggested that the spring-sowing soybeans from the Northeast were classified into MG000-IV, and the summer-sowing soybeans from the Huang-Huai-Hai region and the Northwest were classified into MGII-VI and MGI-III, respectively[4, 54]. In contrast, the spring-sowing and summer-sowing soybean from the Yangtze River region were classified into MG0-IV and MGIII-VIII, respectively. Our data were mostly consistent with previous studies. Unlike the previous data, 11 spring-sowing soybean varieties were classified into MGVII or even later groups in this study, implying that there was rich genetic variations among the Chinese soybean MCC.

In this study, we also classified several accessions such as Dongnong 36, Suinong 14, Jilin 30 and Taixingheidou into the same MG appeared in previous study[4]. For the varieties of Heihe 1 (MG00), Nenfeng 11 (MGI) and Hefeng 25 (MGI), similar MG was registed in United States Department of Agriculture (USDA)[55]. Moreover, MG of some US varieties such as Harosoy, Williams, Clark and Hartwig was in line with the previous study by Chang et al (1992)[56]. Aforementioned results confirmed the experimental methods and the classification of MG. Nonetheless, in this study, Honghuliuyuebao, Duchangwudou and Daqingren were classified into MGII, MGII and MGIV, respectively. However, these 3 varieties were classified into MGI-2, MGI-2, and MGIII-2 by Gai et al (2001)[4]. This might be resulted from the different test conditions and criteria, or various source of accessions.

In North America, the optimal zones for each MG soybeans were roughly parallel with latitude[26]. However, the distribution of MGs in China seems to be relatively complex because of the diversity of ecological condition and production practices. To be exact, the same MG varieties might be from different regions and multiple MGs may present in the same region, which resulted in the abundance of Chinese soybean germplasm resources. The spring-sowing soybeans were mainly classified into MGII-III, whereas, summer- and autumn-sowing soybeans were classified into MGVI-VIII in Jiangxi province.

Previous reports revealed that recessive alleles (e.g. *e1*, *e2*, *e3* and *e4*) were associated with earlier flowering and maturity[15]. In this study, most late-maturing accessions had the same genotype at E1/E2/E3/E4 with 136.2 d of average growth period while most accessions with one or more recessive alleles of *E1*, *E2*, *E3* and *E4* were early-maturing. In line with the previous studies[15, 45, 57] (, the average growth period of accession with single recessive allele was 116.2 d for *e2*, 105.4 d for *e1* and 80.6 d for *e3*, respectively (P<0.05, data not shown). Meanwhile, some varieties with two or more recessive alleles were late maturing. For example, two recessive alleles were identified at *E2* and *E3* loci (*E1/e2-ns/e3-tr/E4*) in Dahuangdou-2 classified into MGIV. The growth period of Dahuangdou-2 was 116.5 d, indicating that there might be other genes controling the flowering and maturity in soybean. These varieties could be used for the discovery of new genes. Moreover, the effects of *E* genes under vegetative periods varied from those under reproductive periods during the soybean development. Previous studies revealed the positive effects of *E1* allele on length of soybean vegetative periods[56, 57](Chang, 1992; Wang et al. 2008). Based on the analysis of allelic variation effects on R/V in this study, we showed the presence of recessive allele in *E1* locus in all thirteen accessions with higher R/V ratio (>2.0). These data revealed that *e1* had a potential effect on late mature by prolonging the reproductive period during soybean growth. In the current study, most of the US reference accessions showed normal agronomic performance in Jining, Shandong province, despite the fact that a few of the early accessions showed poor growth vigor or symptoms of mosaic virus disease. However, more standard reference varieties with the resistance to lodging and disease are required in order to avoid the potential effects of disadvantageous traits on the maturity performances[58–61] (). Hence, the elite accessions with desirable agronomic traits are recommended for the establishing Chinese maturity standard classification system.

## Conclusion

A large spectrum of maturity groups (MG000-MGIX/X) were found in the Chinese soybean MCC, which reflected the complex ecological conditions and planting patterns in China. The MG000 and MG00 accessions were only distributed in northern Heilongjiang, and the MGIX/X accessions were collected only from south China. Other MGs covered the accessions from different regions and sowing types. Recessive alleles of *E* genes were identified with higher frequency in early-maturing accessions and the dominate alleles were detected always in medium and late-maturing accessions. The diversity of maturity groups and allelic variation of maturity genes in MCC confirmed the representativeness of Chinese soybean MCC. Some accessions could be used for the discovery of new soybean maturity genes. The combination roles of *E* loci could be used to design maturity group for the selection of soybean parents and breeding new varieties adapted to desired cropping systems.
